# Life beyond 5 Years after TAVI: Patients' Perceived Health Status and Long-Term Outcome after Transcatheter Aortic Valve Implantation

**DOI:** 10.1155/2019/4292987

**Published:** 2019-10-01

**Authors:** Marie-Isabel K. Murray, Eileen Hofmann, Roberta De Rosa, Silvia Mas-Peiro, Philipp Seppelt, Thomas Walther, Andreas M. Zeiher, Stephan Fichtlscherer, Mariuca Vasa-Nicotera

**Affiliations:** ^1^Department of Cardiology, University Hospital Frankfurt, Frankfurt am Main, Germany; ^2^Department of Cardiac Surgery, University Hospital Frankfurt, Frankfurt am Main, Germany

## Abstract

**Background:**

Transcatheter aortic valve implantation (TAVI) is currently recommended for patients with severe aortic stenosis at intermediate or high surgical risk. The decision process during TAVI evaluation includes a thorough benefit-risk assessment, and knowledge about long-term benefits and outcomes may improve patients' expectation management.

**Objective:**

To evaluate patients' perceived health status and self-reported long-term outcome more than 5 years after TAVI.

**Methods and Results:**

Demographic and procedure data were obtained from all patients treated with TAVI at our institution from 2006 to 2012. A cross-sectional survey was conducted on the patients alive, measuring health status, including the EQ-5D-5L questionnaire, and clinical outcomes. 103 patients (22.8%) were alive at a median follow-up period of 7 years (5.4–9.8). 99 (96%) of the 103 patients were included in the final analysis. The mean age at follow-up was 86.5 years ± 8.0 years, and 56.6% were female. Almost all patients (93.9%) described an improvement of their quality of life after receiving TAVI. At late follow-up, the mean utility index and EQ-VAS score were 0.80 ± 0.20 and 58.49 ± 11.49, respectively. Mobility was found to be the most frequently reported limitation (85.4%), while anxiety/depression was the least frequently reported limitation (19.8%). With respect to functional class, 64.7% were in New York Heart Association (NYHA) class III or IV, compared to 67.0% prior to TAVI (*p*=0.51). Self-reported long-term outcomes revealed mainly low long-term complication rates. 74 total hospitalizations were reported after TAVI, and among those 43% for cardiovascular reasons. Within cardiovascular rehospitalizations, new pacemaker implantations were the most frequently reported (18.9%), followed by cardiac decompensation and coronary heart disease (15.6%).

**Conclusion:**

The majority of the patients described an improvement of health status after TAVI. More than five years after TAVI, the patients' perceived health status was satisfactory, and the incidence of clinical events and hospitalizations was very low.

## 1. Introduction

Aortic stenosis is the most common valve disease in industrialised countries leading to surgery or catheter intervention [1, 2]. If severe symptomatic aortic stenosis is left untreated, prognosis is poor and mortality is up to 50% one year after onset of symptoms and more than 90% after five years [3]. Transcatheter aortic valve implantation (TAVI) is the standard treatment for patients with severe aortic stenosis at high and excessive risk for surgery [1, 4, 5]. Recently, guidelines were expanded, and TAVI is now also recommended as an alternative procedure to conventional surgery in intermediate-risk patients [6–8]. Clinical trials and registry data have demonstrated high procedural success and a significant improvement of survival [9]. However, comprehensive multimodality and multidisciplinary Heart Team assessment is pivotal to ensure best possible outcomes after TAVI [10]. Before patients are admitted for TAVI, they undergo thorough examinations, including functional and cognitive tests. The results are discussed at a multidisciplinary team conference, and risk-benefit analysis determines if TAVI procedure should be recommended. Additionally, the impact of the procedure on health status after TAVI is of importance to guide the patient-centred decision-making process. In the elderly, the consequences of health status after TAVI may be as or even more important than survival since they often express a preference for quality of life over quantity of life [11, 12]. The EQ-5D-5L questionnaire is a suitable instrument for evaluating patients' health status after TAVI since it is a standardized test which has been used in previous studies [13–15]. In addition to health status, there are limited data on long-term complication rates and hospital readmissions following TAVI. Most studies report only one-year follow-up data with a maximum of 5 years [16–20]. In light of these facts, the main objective of this study was to investigate long-term perceived health status and self-reported outcomes at a minimum of 5 years after TAVI.

## 2. Methods

### 2.1. Study Population and Study Design

Between November 2006 and December 2012, a total of 452 patients with severe symptomatic aortic stenosis received TAVI at our institution. After checking survival status between October 2018 and January 2019, a trained professional contacted the patients still alive by telephone and asked a defined set of questions. In addition to some general health-related questions, the EQ-5D-5L questionnaire was conducted. Furthermore, questions about complications and rehospitalizations after TAVI were asked (A detailed list with all questions can be found in the supplementary data, Table S1 and S2 ([Supplementary-material supplementary-material-1])). All patients alive, who underwent TAVI more than 5 years ago, were included in our study regardless of access route or valve type. Patients were excluded from the study if they were cognitively impaired, unable to speak German, or too sick to answer the questions. No data on baseline health status were available. The study was approved by the local ethics committee of the Goethe University of Frankfurt, and it was conducted in accordance with the Declaration of Helsinki.

### 2.2. Procedure

Design features of the balloon-expandable and self-expanding prosthesis and technical details of the procedure have been previously described [21, 22]. The Edwards bioprosthesis, available in 23 mm, 26 mm, and 29 mm sizes, was implanted using the transfemoral or the transapical approach. The CoreValve prosthesis, available in 26 mm, 29 mm, and 31 mm sizes, was implanted using the transfemoral approach. Three patients received a JenaValve with the transapical approach. Two of them died before the follow-up, and only one patient with a 25 mm size JenaValve was included for further analysis. All procedures were performed under local anaesthesia or general anaesthesia with endotracheal intubation.

### 2.3. Health Status Assessment

Health status was measured with the generic European Quality of Life Five Dimensions Five Levels (EQ-5D-5L) questionnaire (Supplementary data, Table S2). The EQ-5D-5L is a standardized health utility Quality of Life (QoL) instrument and is qualified for measuring health status within an elderly population (EuroQoL Group, Germany) [23]. This descriptive system consists of five domains (mobility, self-care, usual activities, pain/discomfort, and anxiety/depression). Each of these domains is divided into five levels of functioning (5L) indicating no problems (level 1), some problem (level 2), moderate problems (level 3), severe problem (level 4), and extreme problems (level 5). There are 3125 possible health states in the EQ-5D-5L questionnaire and each of them is referred to by a five-digit code. The health states can be converted to a utility score, ranging from −0.446 to 1 (a value of 1 indicating full health, while a value lower than 0 represents a status considered to be worse than death). In this study, health status was assessed using a validated German version of the EQ-5D-5L.

The second part of the EQ-5D includes a visual analogue scale (EQ-VAS), with numeric values from 0 (“worst imaginable health state”) to 100 (“best imaginable health state”) [24].

### 2.4. Statistical Analysis

Descriptive statistics were summarized as mean ± SD for normally distributed continuous variables or otherwise as median and 25th to 75th percentile. Categorical variables are described by frequencies and percentages. Differences in paired samples were tested using the Wilcoxon signed-rank test or paired Student's *t*-test. Categorical variables were compared using the chi-square or Fisher's exact test. Statistical significance was defined at a level of *α* ≤ 0.05. Analysis was performed with SPSS, Version 24 (SPSS Inc., Chicago, IL, USA).

## 3. Results

**Figure 1:**
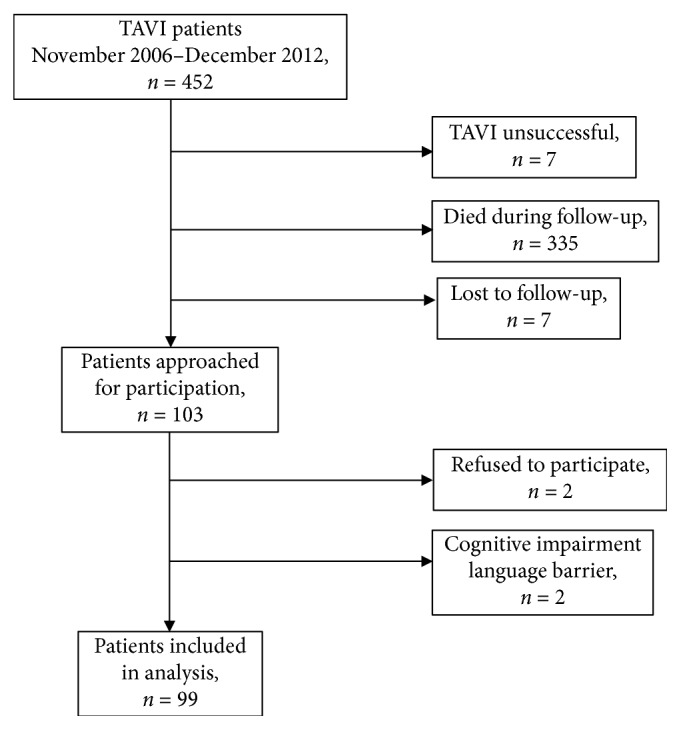
Study flowchart of the number of total TAVI patients between November 2006 and December 2012, and the final study population with more than 5 years follow-up data after TAVI.

From November 2006 to December 2012, 452 patients were consecutively treated with TAVI at our institution. 103 patients (22.8%) were still alive at a median follow-up period of 7 years (5.4–9.8). 335 (74.1%) patients died before the time of inclusion, 7 patients were lost to follow-up, and in 7 cases implantation was not successful (see Figure 1). 99 (96%) of the 103 patients were eligible for the study and agreed to participate in our survey. The mean age at follow-up was 86.5 years ± 8.0 years, and 56.6% were female. Baseline and procedural characteristics are presented in Tables 1 and 2. The devices used were in 58.8% balloon-expandable (Edwards) and in 40.2% self-expanding (CoreValve) prosthetic valves. Only one patient received a self-expanding JenaValve. The transfemoral approach was used in 65.0% of cases and the transapical in the remaining 35%. Early clinical outcome data are depicted in Table 3. In-hospital mortality was 9.2%, the need for a new pacemaker implantation (PPI) was 12.6%, and major bleeding occurred in 5.9% according to VARC-2 criteria [25].

### 3.1. Health Status

Outcomes regarding health status including the EQ-5D-5L results and EQ-VAS scores are listed in Tables 4 and 5. Approximately, two-third (62.7%) of the interviewed patients stated that they are currently in a good general health condition. 93.4% of the patients described an improvement of their health status after receiving TAVI.

With respect to EQ-5D-5L, mobility was found to be the most frequently reported limitation (85.4%), while anxiety/depression was the least frequently reported limitation (19.8%). The majority of the patients had slight to moderate limitations in most domains (Table 5). The mean utility index and EQ-VAS score were 0.80 ± 0.20 and 58.49 ± 11.49, respectively.  Table 4 also shows a comparison of the health status in TAVI patients with the mean values of the age-adjusted German population older than 75 years.

**Figure 2:**
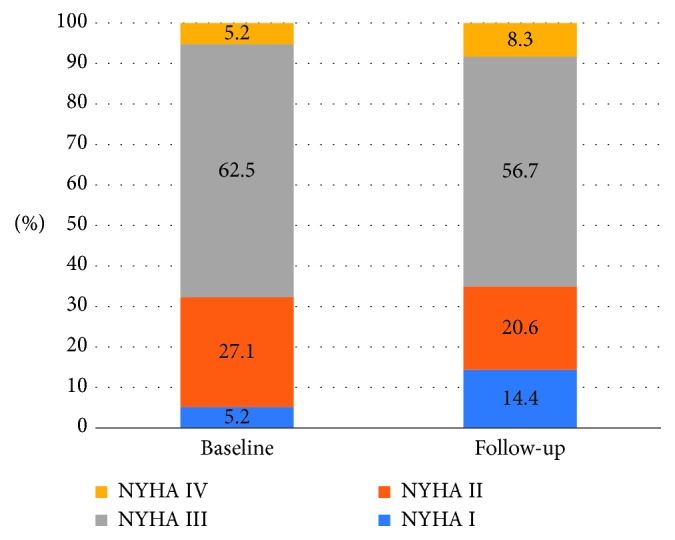
New York Heart Association (NYHA) class at baseline and at follow-up in the survival cohort.

With attention to functional New York Heart Association (NYHA) class, 67.0% were in NYHA class III or IV before TAVI and 64.9% at a median follow-up period of 7 years (*p*=0.51; Figure 2).

### 3.2. Long-Term Outcome and Rehospitalization

**Figure 3:**
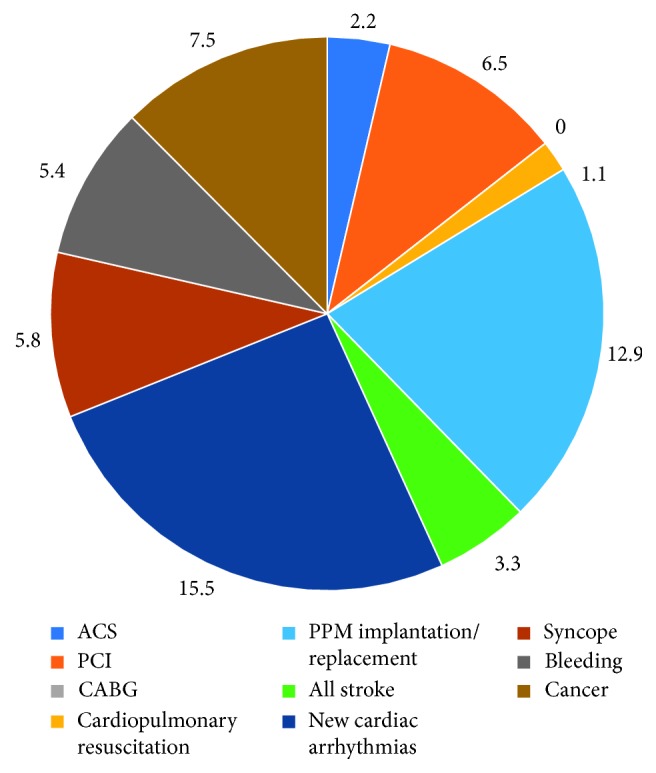
Patient reported long-term outcome after TAVI in percentage (%).

Self-reported long-term outcomes revealed mainly low complication rates (Table 6, Figure 3). All-stroke rate was 3.3%, bleeding occurred in 5.5%, and acute coronary syndrome in 2.2% during the median time of 7 years after TAVI. PPI was necessary in 6 patients, and the overall pacemaker intervention rate including pacemaker replacement was 12.9%. In addition, 15.5% of patients reported events of new cardiac arrhythmias.

Furthermore, patients described 74 total hospitalizations after TAVI, and among them in 43% for cardiovascular reasons. Within cardiovascular rehospitalizations, requirement for PPI was the most frequently reported (18.9%), followed by cardiac decompensation and coronary heart disease (15.6%) (Table 7).

## 4. Discussion

The main finding of the present study analysing a selected group of patients more than five years after TAVI was a satisfactory health status, no significant change in functional (NYHA) class compared to before TAVI, and low self-reported complication and rehospitalization rates. Our study about self-reported health status and outcomes after TAVI is currently the only study with a very long follow-up time with a median duration of 7 years. A high proportion of the patients still alive could be included in the final analysis.

Over 90% of patients described an overall improvement of their health situation after TAVI. With regard to the standardized EQ-5D-5L questionnaire, the current health status showed satisfactory results at late follow-up. Mobility was found to be the most frequent limitation (85.4%), followed by limitation in usual activity (82.3%). Since we did not evaluate baseline EQ-5D-5L, we could not analyse any change in health status. The German TAVI registry evaluated the EQ-5D index after one year and found a significant improvement of health status [13]. Interestingly, however, our results showed a better EQ-5D index after a median follow-up time of 7 years compared to the one-year results of the German TAVI registry (0.80 ± 0.20 vs. 0.70 ± 0.24). In addition, the visual analogue health scale was similar in our study compared to the one-year results of the German TAVI registry (58.49 ± 11.49 vs. 57 ± 19.6). Previous studies focused mainly on the first postprocedural period up to one year after TAVI and most of them showed a substantial improvement in health status [14, 15, 28]. To our knowledge, only one Dutch study published recently data on quality of life data after TAVI with a mean follow-up time of 5.5 years [15]. All patients showed satisfactory quality of life data despite their age and multiple comorbidities. Nevertheless, their study revealed a lower utility score than the result in our analysis (0.69 ± 0.29 vs. 0.80 ± 0.20).

Moreover, our study showed a similar utility score and EQ-VAS score compared with the general age-adjusted German population (Table 4). Of note, the population norms for the EQ-5D were standardized to adults older than 75 years, whereas our patient population had a mean age of 86 years [23].

With respect to NYHA functional class, we revealed no improvement of NYHA class more than 5 years after TAVI, as NYHA class III/IV has been observed in two-third of our patients prior to TAVI and at late follow-up. In contrast to our results, short-term studies have described a sustained improvement of NYHA class in selected groups of survivors [15, 29, 30]. In these reports, most patients were in NYHA class I/II who were in NYHA class III/IV prior to TAVI up to five years after the procedure [31].

These findings may suggest that the initial benefit on functional gain which was described in the first years after TAVI may decline beyond 5 years. However, the advanced age of the population (mean age at follow-up was 86.5 years ± 8.0 years) and concomitant comorbidities may play an important impact on their functional status.

Importantly, all patients alive reported very low incidence of complications in the following years after TAVI. All-stroke rate was 3.3%, which was similar to a previous study published by Barbanti et al. [18]. In this report, neurological event rate was 7.5% at 5 years; however, approximately 5% occurred in the first 6 months after the procedure. Another study by Tarantini et al. observed a stroke rate of 2.5% with the CoreValve and 3.7% with the Edwards Sapiens bioprosthesis at 5 years [30]. In the same report, the incidence of acute coronary syndrome was 2.4% which was in line with our result (2.2%). Recently, data from the FRANCE-2 registry showed that the majority of cardiovascular events occurred in the first months after valve replacement [31]. Our analysis revealed similar outcome data, and most complications including bleeding and pacemaker implementation occurred mainly in the earlier period after TAVI and were described at late follow-up only in 5.4% and 6%, respectively. The high incidence of new cardiac arrhythmias may be due to the fact that the prevalence of many cardiac arrhythmias increases with older age [32, 33]. Larger studies are needed to assess if there is any association between TAVI and late onset of cardiac arrhythmias.

In the present study, there were in total 74 rehospitalizations reported during the median time period of 7 years after TAVI. Cardiovascular reasons accounted for 43.2% with new PPI, heart failure, and coronary heart disease as the most frequent indications. Interestingly, the majority of patients who received a new PPI had a balloon-expandable (four out of six patients) and not self-expanding bioprosthesis. However, larger studies have to evaluate if there is any association between valve type and long-term PPI rate. In general, data on long-term hospital readmissions after TAVI are very limited [16–18, 34]. In a one-year follow-up study, Franzone et al. observed hospital admission in one out of four patients. Cardiovascular rehospitalization was reported in 46.1%, with heart failure as the most frequent reason [34]. Similar to our study, Barbanti et al. observed a rehospitalization rate for cardiovascular reasons in 46% in a 5-year follow-up period [18]. Among all rehospitalizations, acute heart failure was the most frequently reported (42.7%), followed by requirement of permanent pacemaker implantation (17.4%).

### 4.1. Limitations

Our study was performed as a single-centre investigation with a fairly limited number of patients. Second, the study design leads to the exclusion of patients with early death and those who were too ill or cognitively impaired to participate. Selection bias may have occurred. Third, patient self-reported data can be subject to error as the result of a variety of factors, including recall and patients' health knowledge and awareness, possibly leading to underestimation of the true event rates [35–37]. Fourth, medical records or health insurance data may be a better alternative to survey data in order to collect information about health services. However, both data sources also have their limitations. Medical records may be inaccurate due to errors in recording data, and it can be difficult to access them from different hospitals and physicians [38, 39]. Health insurance data do not include health service information from people covered by private insurance or self-payers [40]. Lastly, and most importantly, we did not conduct the survey with the EQ-5D-5L questionnaire before and directly after TAVI. Consequently, we cannot report whether and how much the patients' health status changed from baseline.

## 5. Conclusion

In the present study, about one-fourth of the patients after TAVI survived at a median follow-up period of 7 years. Most of these patients reported about a satisfactory health status at late follow-up. The initial benefits of functional status seemed to be reduced more than 5 years after TAVI. According to the patients' reported outcome, incidence of clinical events and hospitalization was very low in the survival cohort within the first five to ten years after TAVI.

## Figures and Tables

**Table 1:** Baseline characteristics before TAVI and at late follow-up.

Variable	Total (*n* = 103)
*Before TAVI*	
Age, years at TAVI	80.1 ± 7.9 (30–92),
Female, *n* (%)	58 (56.3)
BMI (kg/m^2^)	27.5 ± 4.3
STS score	9.1 ± 5.6
Logistic EuroScore	18.1 ± 11.7
NYHA functional class III to IV	65 (67.0)
Hypertension, *n* (%)	92 (89.3)
Diabetes mellitus, *n* (%)	23 (22.3)
CKD, (GFR <60 ml/min), *n* (%)	63 (61.2)
Previous MI, *n* (%)	19 (18.4)
Previous PCI, *n* (%)	52 (51.5)
Previous CABG, *n* (%)	10 (9.7)
Permanent pacemaker	16 (15.5)
COPD, *n* (%)	15 (14.7)
Prior CVA/TIA	12 (11.6)
Atrial fibrillation/atrial flutter	36 (35.0)

*At late follow-up*	
Age, years	86.5 ± 8.0 (37–98)
Female, *n* (%)	56 (56.6)

Data are expected as absolute values (*n*) and percentages (%) or as mean ± standard deviation (SD). TAVI, transcatheter aortic valve implantation; *n*, number; BMI, body mass index; STS, society of thoracic surgery; NYHA, New York Heart Association; CKD, chronic kidney disease; MI, myocardial infarction; PCI, percutaneous coronary intervention; CABG, coronary artery bypass graft; COPD, chronic obstructive pulmonary disease; CVA; cerebrovascular accident; TIA, transient ischemic attack.

**Table 2:** Procedural characteristics.

Variable	*n* (%)
*Valve type*	
Edward bioprosthesis	60 (58.8)
CoreValve	41 (40.2)
JenaValve	1 (1.0)

*Access, n (%)*	
Transfemoral	68 (65.0)
Transapical	35 (35.0)
Prosthesis after dilatation, *n* (%)	13 (12.7)
Valve-in-valve, *n* (%)	5 (5.0)

*Prosthesis diameter, n (%)*	
23 mm	22 (21.6)
25 mm	1 (1.0)
26 mm	51 (50.0)
29 mm	26 (25.5)
31 mm	2 (2.0)

*Anaesthesia*	
Local	64 (62.1)
General	39 (37.9)

Data are expected as absolute values (*n*) and percentages (%).

**Table 3:** Early clinical outcomes.

Variable	*n* (%)
*Procedural and in-hospital outcomes*	
In-hospital mortality	44 (9.2)
New-onset left bundle branch block	21 (20.4)
Need for pacemaker implantation	13 (12.6)
New-onset atrial fibrillation or flatter post-TAVI	13 (12.6)
Coronary obstruction	0 (0)
Ventricular perforation with tamponade	3 (2.9)
Need for second valve	1 (1.0)
Stroke, *n* (%)	1 (1)
Major vascular complication, *n* (%)^*∗*^	2 (1.9)
Minor vascular complication, *n* (%)^*∗*^	13 (12.7)

*Bleeding, n (%)* ^*∗*^	
Major^*∗*^	6 (5.8)

*AKI, n (%)* ^*∗*^	
Stage 1	2 (1.9)
Stage 2	3 (2.9)
Stage 3	2 (1.9)

Data are expressed as absolute values (*n*) and percentages (%). AKI, acute kidney injury; ^*∗*^as defined in the VARC-2 criteria, valve academic research consortium.

**Table 4:** Health status.

Variable	TAVI patients (*n* = 99)	German population^*∗*^
*Current general health condition*		
Good	62 (62.6)	
Okay	31 (31.3)	
Not good	6 (6.0)	
Better after TAVI	93 (93.9)	

*EQ-5D (% of patients indicating a problem)*		
Mobility	85.4%	54.2%
Self-care	26.1%	16.0%
Usual activities	82.3%	33.8%
Pain/discomfort	52.1%	52.2%
Anxiety/depression	19.8%	6.6%
Utility score	0.80 ± 0.20	0.84 ± 0.14
VAS	58.49 ± 11.49	60.5 ± 20.3

NYHA functional class III to IV	56 (58.3)	
NYHA function ≤III	40 (41.7)	

Data are expected as absolute values (*n*) and percentages (%) or as mean ± SD. TAVI, transcatheter aortic valve implantation; AP, angina pectoris; NYHA, New York Heart Association. ^*∗*^German population norms for the EQ-5D are stratified by age >75 years [26, 27]. EQ-5D, EuroQoL 5 dimensions; VAS, visual analogue score.

**Table 5:** Frequency of patients reporting problems in EQ-5D-3L domains at late follow-up.

	Mobility	Self-care	Usual activities	Pain/discomfort	Anxiety/depression
No problems	14 (14.6)	71 (74.0)	17 (17.7)	46 (47.9)	77 (80.2)
Slight to moderate problems	57 (59.4)	19 (19.8)	71 (74.0)	48 (50.0)	19 (19.8)
Severe to extreme problems	25 (26.0)	6 (6.3)	8 (8.3)	2 (2.1)	0 (0)

Data are expected as absolute values (*n*) and percentages (%).

**Table 6:** Self-reported long-term outcome.

Variable	*n* (%)
Hospitalization	74 (78.7)
Hospitalization for CVD	32 (31.1)
ACS	2 (2.2)
PCI	6 (6.5)
CABG	0 (0)
Cardiopulmonary resuscitation	1 (1.1)
PP implantation or replacement	12 (12.9)
All stroke	3 (3.3)
New cardiac arrhythmias	16 (15.5)
Syncope	6 (5.8)
Bleeding minor	5 (5.4)
Bleeding major	0 (0)
Cancer	7 (7.5)

Data are expected as absolute values (*n*) and percentages (%). CVD, cardiovascular disease; ACS, acute coronary syndrome; PCI, percutaneous coronary intervention; CABG, coronary artery bypass graft; PP, permanent pacemaker.

**Table 7:** Reasons for cardiovascular disease hospitalization.

Variable	*n* (%)
Cardiac decompensation	5 (15.6)
Coronary heart disease	5 (15.6)
New pacemaker implantation (4 ES, 1 CV, 1 unknown)	6 (18.8)
Pacemaker replacement	4 (12.5)
Prior CVA/TIA	3 (9.4)
Mitral valve surgery	2 (6.3)
Reoperation of aortic valve	2 (6.3)
Cardiac arrhythmias	3 (9.4)
Peripheral arterial disease	1 (3.1)
ICD implantation	1 (3.1)
Left atrial appendage closure	1 (3.1)

Data are expected as absolute values (*n*) and percentages (%). ES, Edwards sapiens; CV, CoreValve; CVA, cerebrovascular accident; TIA, transient ischemic attack; ICD, implantable cardioverter-defibrillator.

## Data Availability

The survey data used to support the findings of this study are available from the corresponding author upon request.

## Supplementary Materials

Supplementary Materials Table S1: questionnaire. Table S2: EQ-5D-5L questionnaire.
