# Supplementary material for: Life beyond 5 Years after TAVI: Patients' Perceived Health Status and Long-Term Outcome after Transcatheter Aortic Valve Implantation
Source: J Interv Cardiol. 2019 Oct 1;2019:4292987. doi: 10.1155/2019/4292987 (PMC6794985; doi:10.1155/2019/4292987)

Supplementary Material

Table S1. Questionnaire

Questions:	Possible Answers:
1. How is your **general health condition**?	Good- okay- bad
1.1. Has your **well-being improved** after TAVI?	Yes - No
1.2. How many **flights of stairs** can you walk without problems?	0-1-2-3-4
2. Have you been **admitted to a hospital** after TAVI?	Yes- No
2.1. If yes, please indicate the **exact reason for hospital admission.**	
3. Have you had any of the **following events** after TAVI:	
3.1. Myocardial infarct	Yes- No
3.2. Cardiac catheter or stent implantation	Yes- No
3.3. Bypass operation	Yes- No
3.4. Cardiopulmonary resuscitation	Yes- No
3.5. Pacemaker implantation	Yes- No
3.6. Defibrillator implantation	Yes- No
3.7. Stroke	Yes- No
3.8. New cardiac arrhythmia	Yes- No
3.9. Syncope	Yes- No
3.10. Bleeding (major or minor)	Yes- No
3.11. Cancer	Yes- No

Table 2. EQ-5D-5L Questionnaire

Under each heading, please check the **ONE** box that best describes your health **TODAY**

Mobility

I have no problems walking	☐
I have slight problems walking	☐
I have moderate problems walking	☐
I have severe problems walking	☐
I am unable to walk	☐

Self-care

I have no problems washing or dressing myself	☐
I have slight problemswashing or dressing myself	☐
I have moderate problems washing or dressing myself	☐
I have severe problems washing or dressing myself	☐
I am unable to wash or dress myself	☐

Usual activities *(e.g. work, study, housework, family or leisure activities)*

I have no problems doing my usual activities	☐
I have slight problems doing my usual activities	☐
I have moderate problems doing my usual activities	☐
I have severe problems doing my usual activities	☐
I am unable to do my usual activities	☐

Pain/discomfort

I have no pain or discomfort	☐
I have slight pain or discomfort	☐
I have moderate pain or discomfort	☐
I have severe pain or discomfort	☐
I am extreme pain or discomfort	☐

Anxiety/ depression

I am not anxious or depressed ☐

I am slight anxious or depressed ☐

I am moderate anxious or depressed ☐

I am severe anxious or depressed ☐

I am extremely anxious or depressed ☐

- We would like to know how good or bad your health is **TODAY**
- This scale is numbered from **0 to 100**
- **100** means the best health you can image

  0 means the worst health you can imagine
- Mark an **X** on the scale to indicate how your health is **TODAY**

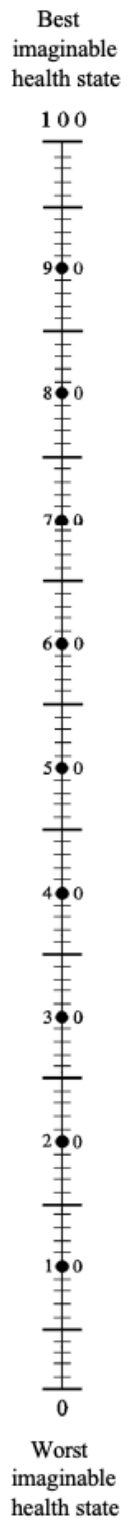

Supplement: Supplementary Materials — Table S1: questionnaire. Table S2: EQ-5D-5L questionnaire. [file 4292987.f1.pdf]
